# Supplementary material for: Unusual Findings of Human-Associated Four-Nucleated Entamoeba Species in Captive Wild Animals
Source: Animals (Basel). 2025 Jan 3;15(1):90. doi: 10.3390/ani15010090 (PMC11718783; doi:10.3390/ani15010090)

Supplemental file S1: Chromatogram obtained after sequencing the PCR amplicon from mandrill samples with primer E12D (see main text for details). The chromatogram displays double peaks; the same fragment is interpreted to show the peaks corresponding to the sequence of *Entamoeba nuttalli* PQ389465 (upper image) and the sequence of *Entamoeba hartmanni* PQ389466 (lower image). The complete sequences (text version) are shown above, presented side by side, unaligned, to indicate the bases that would be observed as overlapping at each position in the chromatogram. The region displayed in the chromatogram is marked in both sequences.

```
E.nuttalli (Mandrill) PQ389465     GATTAAGAGTTCTTTCATGATTTATTGGGTAGTGGTGCATGGCCGTTCTTAGTTGGTGGAGTGATTTGTCAGGTTAATTCCGGTAACGAACGAGACTGAA
E.hartmanni(Mandrill) PQ389466     GATTAAGAGATCTTTCATGATTTAATGGTGAGTGGTGCATGGCCGTTCTTAGTTGGTGGTATGAATTGTCAGGTTAATTCCGGTAACGAACGAGACTGAA

E.nuttalli (Mandrill) PQ389465     ACCTATTAATTAGTTTTCTGCCTATAAGACAGAAATGTTCGCAAGAACAGGTGCGTAAGTACCACTTCTTAAAGGGACACATTTCAATTGTCCTATTTTA
E.hartmanni(Mandrill) PQ389466     ACCTATTAATTAGTTCTGTGCCTATAAGACACAGGTGTTAGCAATAACAGATAGCGATATCACTTCTTAAAGGGACACATTTCAAATGTTCTTTGATTAT

E.nuttalli (Mandrill) PQ389465     ATTGTAGTTATCTAATTTCGGTTAGAGCTCTTTTAACGTGGGAAAAAGAAAAAGGAAGCATTCAGCAATAACAGGTCTGTGATGCCCTTAGACATCTTGG
E.hartmanni(Mandrill) PQ389466     CAAAAGTTGGATTCTGTCCAACGTGATAAACGAAGAAAAAAGAAACAGGAAGCATTCAGCAATAACAGGTCTGTGATGCCCTTAGACATCTTGGGCTGCA

E.nuttalli (Mandrill) PQ389465     GCCGCACGCGCGCTACAATGGAGTTACTAGAGAGCATTTTATCATTTACACCTTATTTATTAGGCTTTGTCTAATAATTAAGGATAGTGAGTGGTGTACC
E.hartmanni(Mandrill) PQ389466     CGCGCGCTACAATGAAGATAGTAGAGAGTAATATATATATGACAGTTTCGTAATCATATATTGTTTACAATGTTGGTTATAGAAAGTGTCATAAACCGAA

E.nuttalli (Mandrill) PQ389465     GAGATTGAAATAGTTAAGGAAAACTCAAAAGAACGTACATGACAGGGATAAATGATTGGAATTATTTGTTTTGAACGAGGAATTCCTTGTAATATCGAGT
E.hartmanni(Mandrill) PQ389466     ACCGTCAGGTGGAGGAAAACTTGAAGCATCGTACATGACTGGGATAAATGATTGGAATTATTTGTTTTGAACGAGGAATTCCTTGTAATATCACGTCATC

E.nuttalli (Mandrill) PQ389465     CATTAACTCGAGATGAATACGTCCCTGCCCTTTGTACACACCGCCCGTCGCTCCTACCGATTGAATAAAGAGGTGAAATTCTAGGATTCTGTCTTATAGA
E.hartmanni(Mandrill) PQ389466     AACGTGAGATGAATACGTCCCTGCCCTTTGTACACACCGCCCGTCGCTCCTACCGATTGAATAAAGAGGTGAAATTCTAAGAGTCAAGTAATTCTGTTTA

E.nuttalli (Mandrill) PQ389465     TAGAAAAATGGATTTAAATCTCCTTATTTAGAGGAAGGAGAAGTCGTAACAAGGTTTCCGTAGGTGAACCTGCGGAAGGATCAA
E.hartmanni(Mandrill) PQ389466     TTTGACAAATGGATTTAAATCTCCTTATTTAGAGGAAGGAGAAGTCGTAACAAGGTTTCCGTAGG
```

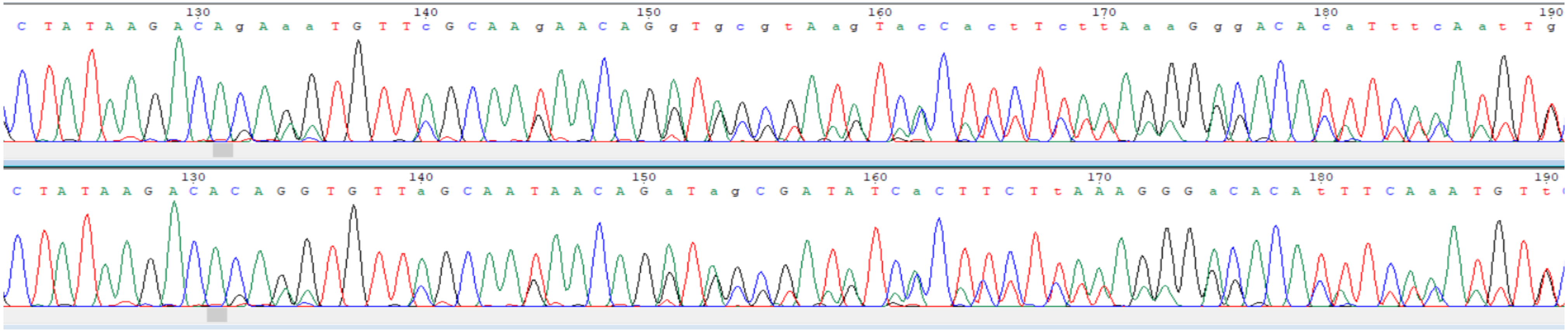

Supplement: Supplementary file 1 [file animals-15-00090-s001.zip › Supplementary file S1.pdf]
