# Supplementary material for: Unusual Findings of Human-Associated Four-Nucleated Entamoeba Species in Captive Wild Animals
Source: Animals (Basel). 2025 Jan 3;15(1):90. doi: 10.3390/ani15010090 (PMC11718783; doi:10.3390/ani15010090)
Supplement: Supplementary file 1 [file animals-15-00090-s001.zip › Supplementary file S2.pdf]

Supplementary file S2: Complete list of missidentified *Entamoeba nuttalli* sequences available in GenBank as of 21/04/2024. These sequences are identified as *Entamoeba histolytica*, but they most likely correspond to *E. nuttalli*. Only sequences AB197936 (Takano et al., *Parasitol Res* **2007**, *101*, 539–546, DOI 10.1007/s00436-007-0510-2) and AB426549 (Suzuki et al., *J Zoo Wildl Med* **2007**, *38*, 471–474, DOI 10.1638/2006-0068.1) (in bold) have been published; the remaining sequences were listed as unpublished in GenBank at the time of this writing.

**Accession number**	**Host**	% identity (BASTn) with *Entamoeba nuttalli* **(AB282657)**	% identity (BASTn) with *Entamoeba histolytica* **(X65163)**
**AB197936**	Cynomolgus monkey	1628/1630 (99.88%)	1617/1630 (99.20%)
**AB426549**	De Brazza's guenon	1628/1630 (99.82%)	1617/1631 (99.14%)
KP233837	Human	1628/1631 (99.82%)	1617/1631 (99.14%)
MK332025	El-Rahawy Drain	1628/1631 (99.82%)	1617/1631 (99.14%)
MT296770	¿?	806/807 (99.88%)	799/807 (99.01%)
MT296771	¿?	805/807 (99.75%)	798/807 (98.88%)
MT296772	¿?	807/807 (100%)	800/807 (99.13%)
MT296773	¿?	804/807 (99.63%)	797/807 (98.76%)
MT296774	¿?	805/807 (99.75%)	798/807 (98.88%)
MT296775	¿?	806/807 (99.88%)	799/807 (99.01%)
MT296776	¿?	806/807 (99.88%)	799/807 (99.01%)
MT296777	¿?	806/807 (99.88%)	799/807 (99.01%)
MT296778	¿?	806/807 (99.88%)	799/807 (99.01%)
MT296779	¿?	804/807 (99.63%)	797/807 (98.76%)
MW426045	Human	759/762 (99.61%)	752/762 (98.69%)
MW426046	Human	759/760 (99.87%)	752/760 (98.95%)
MW426047	Human	762/763 (99.87%)	755/763 (98.95%)
MW426048	Human	759/760 (99.87%)	752/760 (98.95%)
MW426051	Human	755/757 (99.74%)	748/757 (98.81%)
MW426055	Rat	760/762 (99.74%)	753/762 (98.82%)
MW426056	Rat	758/760 (99.74%)	751/760 (98.82%)
MW426057	Rat	755/757 (99.74%)	748/757 (98.81%)
MW426058	Rat	755/758 (99.60%)	748/758 (98.68%)
MW426059	Rat	758/760 (99.74%)	751/760 (98.82%)
MW426060	Rat	755/757 (99.74%)	748/757 (98.81%)
MW426065	Cattle	747/748 (99.87%)	740/748 (98.63%)
MW426070	Sheep	753/757 (99.47%)	746/757 (98.55%)
MW426073	Sheep	755/756 (99.87%)	748/756 (98.94%)
MW440565	Human	417/418 (99.76%)	415/418 (99.28%)
MW440566	Human	417/418 (99.76%)	415/418 (99.28%)
MW440567	Human	416/418 (99.52%)	414/418 (99.04%)
MW440568	Human	417/418 (99.76%)	415/418 (99.28%)
MW440569	Human	417/418 (99.76%)	415/418 (99.28%)
MW440570	Human	417/418 (99.76%)	415/418 (99.28%)
MW440571	Human	416/418 (99.52%)	414/418 (99.04%)
MW440572	Human	417/418 (99.76%)	415/418 (99.28%)
MW440573	Human	417/418 (99.76%)	415/418 (99.28%)
MW440574	Human	416/418 (99.52%)	414/418 (99.04%)

MW440575	Cattle	417/418 (99.76%)	415/418 (99.28%)
MW440576	Cattle	417/418 (99.76%)	415/418 (99.28%)
MW440577	Cattle	417/418 (99.76%)	415/418 (99.28%)
MW440578	Cattle	416/418 (99.52%)	414/418 (99.04%)
MW440579	Cattle	416/418 (99.52%)	414/418 (99.04%)
MW440580	Cattle	418/418 (100%)	416/418 (99.52%)
MW440581	Cattle	418/418 (100%)	416/418 (99.52%)
MW440583	Cattle	414/418 (99.04%)	412/418 (98.56%)
MW440584	Cattle	416/418 (99.52%)	414/418 (99.04%)
MZ377020	Water	427/427 (100%)	424/427 (99.30%)
MZ377021	Water	425/425 (100%)	422/425 (99.29%)
OM268853	Human	467/467 (100%)	463/467 (99.14%)
OM268854	Human	461/461 (100%)	457/461 (99.13%)
OM268855	Human	448/448 (100%)	444/448 (99.11%)
OM268857	Human	467/467 (100%)	463/467 (99.14%)
OM268862	Human	461/461 (100%)	457/461 (99.13%)
ON086990	Human	608/609 (99.84%)	603/609 (99.01%)
ON086991	Human	605/606 (99.83%)	600/606 (99.01%)
ON086992	Human	608/609 (99.84%)	603/609 (99.01%)
ON724165	Cattle	455/455 (100%)	451/455 (99.12%)
ON724167	Cattle	448/448 (100%)	444/448 (99.11%)
ON724169	Cattle	448/448 (100%)	444/448 (99.11%)
OP451868	Human	448/448 (100%)	444/448 (99.11%)
OP454049	Human and domestic dogs	455/455 (100%)	451/455 (99.12%)
OP456534	Human and monkey	806/807 (99.88%)	799/807 (99.01%)
OP456535	Human and monkey	805/807 (99.75%)	798/807 (98.88%)
OP456536	Human and monkey	807/807 (100%)	800/807 (99.13%)
OP456537	Human and monkey	804/807 (99.63%)	797/807 (98.76%)
OP456538	Human and monkey	805/807 (99.75%)	798/807 (98.88%)
OP456539	Human and monkey	806/807 (99.88%)	799/807 (99.01%)
OP456540	Human and monkey	806/807 (99.88%)	799/807 (99.01%)
OP456541	Human and monkey	806/807 (99.88%)	799/807 (99.01%)
OP456542	Human and monkey	806/807 (99.88%)	799/807 (99.01%)
OP456543	Human and monkey	804/807 (99.63%)	797/807 (98.76%)
OP476328	Human and domestic dogs	806/80 (99.88%)	799/807 (99.01%)
OP476329	Human and domestic dogs	805/807 (99.75%)	798/807 (98.88%)
OP476330	Human and domestic dogs	807/807 (100%)	800/807 (99.13%)
OP476331	Human and domestic dogs	804/807 (99.63%)	797/807 (98.76%)
OP476332	Human and domestic dogs	805/807 (99.75%)	798/807 (98.88%)
OP522013	Monkey	806/807 (99.88%)	799/807 (99.01%)
OP522014	Monkey	806/807 (99.88%)	799/807 (99.01%)
OP522015	Monkey	806/807 (99.88%)	799/807 (99.01%)
OP522016	Monkey	806/807 (99.88%)	799/807 (99.01%)
OP522017	Monkey	804/807 (99.63%)	797/807 (98.76%)
OP522416	Domestic dogs	806/807 (99.88%)	799/807 (99.01%)
OP522417	Domestic dogs	805/807 (99.75%)	798/807 (98.88%)
OP522418	Domestic dogs	807/807 (100%)	800/807 (99.13%)
OP522419	Domestic dogs	804/807 (99.63%)	797/807 (98.76%)

OP522420	Domestic dogs	805/807 (99.75%)	798/807 (98.88%)
OP522421	Domestic dogs	806/807 (99.88%)	799/807 (99.01%)
OP522422	Domestic dogs	806/807 (99.88%)	799/807 (99.01%)
OP522423	Domestic dogs	806/807 (99.88%)	799/807 (99.01%)
OP522424	Domestic dogs	806/807 (99.88%)	799/807 (99.01%)
OP522425	Domestic dogs	804/807 (99.63%)	797/807 (98.76%)
OP526382	Human	455/455 (100%)	451/455 (99.12%)
OP363363	¿¿??	566/566 (100%)	561/566 (99.12%)
OP363364	¿¿??	567/567 (100%)	562/567 (99.12%)
OP363365	¿¿??	565/565 (100%)	560/565 (99.12%)
OP363366	¿¿??	566/566 (100%)	561/566 (99.12%)
OP537201	Domestic dogs	806/807 (99.88%)	799/807 (99.01%)
OP537202	Domestic dogs	805/807 (99.75%)	798/807 (98.88%)
OP537203	Domestic dogs	807/807 (100%)	800/807 (99.13%)
OP537204	Domestic dogs	804/807 (99.63%)	797/807 (98.76%)
OP537205	Domestic dogs	805/807 (99.75%)	798/807 (98.88%)
OP626161	Monkeys	806/807 (99.88%)	799/807 (99.01%)
OP626162	Monkeys	805/807 (99.75%)	798/807 (98.88%)
OP626163	Monkeys	807/807 (100%)	800/807 (99.13%)
OP626164	Monkeys	804/807 (99.63%)	797/807 (98.76%)
OP626165	Monkeys	805/807 (99.75%)	798/807 (98.88%)
OP808369	Domestic dogs	1628/1631 (99.82%)	1617/1631 (99.14%)
OQ880536	Human	418/418 (100%)	414/418 (99.04%)
OQ880537	Human	426/426 (100%)	422/426 (99.06%)
